# Differences in bone histomorphometry between White postmenopausal women with and without atypical femoral fracture after long-term bisphosphonate therapy

**DOI:** 10.1093/jbmr/zjae018

**Published:** 2024-02-05

**Authors:** Shijing Qiu, Ruban Dhaliwal, George Divine, Elizabeth Warner, Sudhaker D Rao

**Affiliations:** Bone and Mineral Research Laboratory, Henry Ford Health, Detroit, MI 48202, USA; Center for Mineral Metabolism and Clinical Research, The University of Texas Southwestern Medical Center, Dallas, TX 75390, USA; Division of Endocrinology, Department of Internal Medicine, The University of Texas Southwestern Medical Center, Dallas, TX 75390, USA; Department of Public Health Sciences, Henry Ford Health, Detroit, MI 48202, USA; Division of Endocrinology, Diabetes, and Bone & Mineral Disorders, Henry Ford Health, Detroit, MI, 48202, USA and Michigan State University College of Human Medicine, East Lansing, MI, 48825, USA; Bone and Mineral Research Laboratory, Henry Ford Health, Detroit, MI 48202, USA; Division of Endocrinology, Diabetes, and Bone & Mineral Disorders, Henry Ford Health, Detroit, MI, 48202, USA and Michigan State University College of Human Medicine, East Lansing, MI, 48825, USA

**Keywords:** atypical femoral fracture, bisphosphonates, bone histomorphometry, wall thickness, turnover, mineralizing surface, osteoporosis

## Abstract

Bone histomorphometric endpoints in transilial biopsies may be associated with an increased risk of atypical femoral fracture (AFF) in patients with osteoporosis who take antiresorptives, including bisphosphonates (BPs). One way to test this hypothesis is to evaluate bone histomorphometric endpoints in age-, gender-, and treatment time-matched patients who either had AFF or did not have AFF. In this study, we performed transiliac bone biopsies in 52 White postmenopausal women with (*n* = 20) and without (*n* = 32) AFFs, all of whom had been treated for osteoporosis continuously with alendronate for 4–17 yr. Despite the matched range of treatment duration (4–17 yr), AFF patients received alendronate for significantly longer time (10.7 yr) than non-AFF patients (8.0 yr) (*P* = .014). Bone histomorphometric endpoints reflecting microstructure and turnover were assessed in cancellous, intracortical, and endocortical envelopes from transilial biopsy specimens obtained from BP-treated patients 3–6 mo after AFF and from non-AFF patients with similar age-, gender-, and range of BP treatment duration. However, in both cancellous and intracortical envelopes, AFF patients had significantly lower wall thickness (W.Th) and higher osteoclast surface (Oc.S/BS) than non-AFF patients. In addition, AFF patients had significantly higher eroded surface (ES/BS) only in the intracortical envelope. None of the dynamic variables related to bone formation and turnover differed significantly between the groups. In conclusion, in the ilium of BP-treated patients with osteoporosis, AFF patients have lower thickness of superficial bone (lower W.Th) of the cancellous and cortical envelopes than non-AFF patients. AFF and non-AFF patients have a similar bone turnover rate in the ilium. Furthermore, in this population, as in previous work, AFF is more likely to occur in BP-treated patients with longer treatment duration.

## Lay Summary

Bisphosphonates (BPs) are widely used to prevent osteoporotic fracture and treat osteoporosis. However, prolonged use of BPs may increase the risk of atypical femoral fracture (AFF), and their pathogenesis remains unclear. This study compared the bone histomorphometric findings in cancellous and cortical bones between White osteoporotic women with (*n* = 20) and without AFF (*n* = 32), who had received BP treatment for a matched duration of 4–17 yr. The BP-treated patients with AFF had significantly lower wall thickness (W.Th) in both cancellous and cortical bones compared to BP-treated patients without AFF. There were no significant differences in bone formation, turnover, or mineral apposition rate between BP-treated AFF and non-AFF patients. In conclusion, our study results suggest that AFF risk is increased in BP-treated patients with smaller young and healthy superficial bone areas (indicated by lower W.Th). Surprisingly, we also discovered that patients with and without AFF have similar bone turnover rates, which contradicts previous beliefs. Our findings provide valuable insights into the potential factors contributing to AFF in BP-treated patients.

## Introduction

Bisphosphonates (BPs), a class of antiresorptive drugs that inhibit osteoclastic bone resorption, are widely used to prevent osteoporotic fracture and treat osteoporosis and other bone diseases.^1-3^ The long-term use of *BPs* increases the risk of atypical femoral fracture (AFF) with reported incidence rates up to 2.4% in specific groups, such as those who continuously take an osteoporosis dose of anti-resorptive for at least 8 yr and those who take an oncology dose of anti-resorptive for at least 1 yr.^4-13^ We observed 450 women with osteoporosis who received BP treatment for an average of 6.0 ± 4.2 yr, and we found that the prevalence of AFF was 4.4%. The prevalence of AFF was 7.5% in Asians, 5.4% in Whites, and 1.8% in Blacks (unpublished data). The incidence of AFF is positively associated with the prevalence of BP use and duration of BP exposure.^6^^,^^14^^,^^15^ Additionally, healing of AFF may be delayed in patients continuing BP treatment.^16^ These serious life-changing complications can result in a high morbidity and lower quality of life. As a result, BP prescription rates have declined greatly because of the patients’ concerns about AFF.^14^^,^^17-19^

The pathogenesis of AFF is complex and not fully understood.^3^^,^^20^ Earlier studies showed that AFF patients had significantly lower activation frequency and bone formation rate than healthy premenopausal normal women.^21-24^ Bone histomorphometric data in AFF patients often disclose the presence of severely suppressed bone turnover (SSBT), a condition when activation frequency is below the lower limit of the reference interval for healthy pre-menopausal women.^25-27^ Since it is a consistent finding in AFF patients, a role for SSBT in the development of AFF has been under consideration for some time.^28^ However, past histomorphometric studies of patients taking anti-resorptives show that SSBT is also a frequent finding in osteoporotic patients without AFF who take anti-resorptives.^29-33^ The appropriate comparator group for BP-treated osteoporotic women with AFF is osteoporotic women treated with BPs for a similar duration without developing AFF. To the best of our knowledge, no such study has been reported as of yet.

Dual-energy X-ray absorptiometry and biochemical bone turnover markers are widely used, respectively, to assess the BMD and the overall bone turnover status of the skeleton to estimate the risk of osteoporotic fracture and monitor treatment efficacy.^34-36^ However, neither tool provides information about bone microarchitecture as well as bone turnover and mineralization at the cellular and tissue levels. Bone histomorphometry is the only technique to do so.^37-39^ Thus, bone histomorphometric outcomes are important for the evaluation of long-term effects of therapeutic agents on bone quality and turnover. To the best of our knowledge, there is very little or no information regarding the differences in comprehensive bone histomorphometry between AFF and non-AFF patients on long-term BP therapy, which was the objective of this study. To achieve this goal, we compared the detailed bone histomorphometric findings in cancellous and cortical bone between BP-treated patients with and without AFF with the same range of BP exposure period (4–17 yr). This study is exploratory in nature which may generate new hypotheses and theories on the pathogenesis of AFF.

## Materials and methods

### Subjects

Eighty-one postmenopausal women with osteoporosis treated with long-term BP therapy (>2 yr) were recruited in this study between 2014 and 2018. The BP duration was determined by patient recall supported by medical record/report review to the nearest mo. Subjects were part of a larger group of 801 patients designed to determine the prevalence of AFF. Of all 801 patients, 450 received BP treatment. In these BP-treated patients, iliac bone biopsy was obtained from 57 without AFF (45 Whites and 12 Blacks) and 20 with AFF (16 Whites, 2 Blacks, and 2 Asians). We compared the histomorphometric data between Black and White women without AFF and found that the values of some variables were significantly different between them. In this study, we included 20 White patients with AFF, of whom 16 were from the 450 BP-treated patients and the other 4 were from our previous study that used the same protocol for observation and analysis.

A control group (32 cases) was selected from 45 White non-AFF patients whose BP durations were within the range of BP durations in the 20 White AFF patients. Thus, there were 32 non-AFF patients and 20 AFF patients with matched range of BP duration in this study. All recruited patients were treated with alendronate 10 mg/d or 70 mg/wk, in addition to total daily intake of 1000 mg of calcium and 400–800 IU of vitamin D. None of the patients were on corticosteroids or other medications known to affect bone turnover as required by the parent study.

Antero-posterior radiographs of the femurs either by Single Energy Femur (Hologic, Marlborough, MA, United States) or by conventional radiography were obtained for all patients to confirm the diagnosis of AFF and for group assignment. Incomplete AFFs were confirmed by MRI, nuclear scintigraphy, or digital tomosynthesis as necessary.^40^ Both complete and incomplete AFFs were assigned to AFF group. The diagnosis of AFF was designated in accordance with the criteria recommended by the Task Force Report of the American Society for Bone and Mineral Research.^20^^,^^41^ The time interval between AFF diagnosis and bone biopsy was 3–6 mo in all patients.

### Bone histomorphometry

All patients received in vivo double tetracycline labeling with an inter-label time of 14 d: the schedule was oxytetracycline, 250 mg every 8 hours for 3 d, followed by an 11-d interval, then demethylchlor-tetracycline, 150 mg every 8 hours for 3 d, finishing 4 d before the biopsy.^42^ A full-thickness transiliac bone biopsy with 2 intact cortices was obtained with a 7.5-mm Gauthier trephine from each patient. The specimen was processed, embedded, sectioned, stained, and examined as previously reported.^25^^,^^43^^,^^44^ In non-AFF patients, the biopsy was obtained from the right ilium. In patients with 1-side AFF, the biopsy was obtained from the contralateral ilium. In patients with bilateral AFFs, the biopsy was obtained from the ilium on the side of the older AFF. To reduce the variability in sample procurement, all biopsies were performed by a single operator (S.D.R.). Standard bone histomorphometric measurements were performed by a histotechnologist blinded to the patients’ AFF status. The bone histomorphometric variables were designated in accordance with the nomenclature recommended by the American Society for Bone and Mineral Research.^38^

The measurements were performed in cancellous, intracortical, and endocortical bone envelopes. The static histomorphometric indices were measured in sections stained with modified toluidine blue method; the dynamic indices were measured in the unstained sections. All measurements were performed using a Bioquant image analyzer equipped with bright-field and fluorescent microscopes.^24^^,^^25^ The parameters related to bone structure included total bone volume per tissue volume (BV/TV, %), trabecular thickness (Tb.Th, μm) and number (Tb.N, #/mm2), and cortical thickness (Ct.Th, μm). The static indices included osteoid and eroded surfaces as a fraction of bone surface (OS/BS, % and ES/BS, %), wall thickness (W.Th, μm), and osteoid thickness (O.Th, μm). The surface lengths covered by osteoblasts and osteoclasts (Ob.S and Oc.S) were expressed as a fraction of bone surface (Ob.S/BS, % and Oc.S/BS, %) respectively.

The dynamic indices were measured based on tetracycline labeling. The extent of bone mineralizing surface (MS) was labeled by double or single tetracycline labeling, from which the MS as a fraction of total bone surface (MS/BS, %) was calculated. The surface lengths (mm) covered by the first label (Lb1.S) and second label (Lb2.S) were measured. The MS calculated by the formula (=(Lb1.S + Lb2.S)/2) yields similar results to the formula of (=dLS+(0.5*sLS)). Mineral apposition rate (MAR, μm/d) was obtained from the average distance between the 2 tetracycline labels divided by the interval of administration (14 d in our study). Bone formation rate at the surface level (BFR/BS, μm^3^/μm^2^/yr) was calculated as MAR*(MS/BS). Activation frequency (Ac.f, #/yr), the annual probability of activation of a new remodeling site at any given locus on the bone surfaces, was derived from (BFR/BS)/W.Th. For the specimens containing only single label, a minimum value of 0.3 μm/d was assigned to MAR, and if there was no label, the MAR was treated as a missing value, and MS/BS, BFR/BS, and Ac.f were assigned a value of 0.^24^^,^^45^ The distance-based values (W.Th, O.Th, and MAR) were corrected for section obliquity by multiplying by π/4. All recommendations for tissue area and surface minimums were followed.^38^

The SSBT in each bone envelopes was determined by the levels of Ac.f, which ranged from 0 to the value below the lower bound of the reference interval for healthy pre-menopausal women.^25^

### Statistical analysis

Mann–Whitney test was used to compare the differences in continuous variables (age, duration of BP exposure, and histomorphometric variable) between BP-treated patients with and without AFF. The proportional data between patients with and without AFF were compared using Fisher’s exact test. Results were considered to be statistically significant if *P* < .05. The analyses were not adjusted for multiple comparisons, so the statistical significance level was maintained at *P* < .05.^46^^,^^47^ Given the potential for type I error due to multiple comparisons, the findings should be interpreted as exploratory.^48^^,^^49^

## Results

### Demographic and clinical characteristics

The demographic information is presented in Table 1. TransIliac bone biopsies were obtained from 52 White postmenopausal women (20 with and 32 without AFF), with the 2 groups having matched range of BP duration (4–17 yr). There was no significant difference in mean age between patients with and without AFF (67.5 ± 5.75 vs 67.9 ± 6.35 yr, *P* = .992). However, the mean duration of BP treatment was significantly longer in AFF patients than in non-AFF patients (10.7 ± 3.93 vs 8.00 ± 3.49 yr, *P* = .014) due to the opposite direction of skewness of BP duration in the 2 groups. The BP duration in non-AFF group was positively skewed (skewness = 0.945). By contrast, BP duration in AFF group was negatively skewed (skewness = -0.049). The proportion of patients treated with BP > 8 yr was significantly higher in the AFF than in the non-AFF (80% vs 50%, *P* < .05) group.

**Table 1:** Demographic characteristics of patients with and without AFF.

	Non-AFF	AFF	*P*
Number of patients	32	20	
Sex	All female		
Menses status	All postmenopause		
Race	All White		
Age (yr)	67.9 (6.35)	67.5 (5.75)	.992
BP exposure (yr)	8.00 (3.49)	10.7 (3.93)	.014
Range of BP exposure (yr)	4–17	4–17	
Skewness of BP exposure	0.945	−0.049	
BP exposure <8 yr	16 (50%)	4 (20%)	.042
BP exposure *>*8 yr	16 (50%)	16 (80%)	

Data expressed as mean (SD) and number (percent). AFF, atypical femoral fracture; BP, bisphosphonate.

### Bone histomorphometry

Bone histomorphometric data for cancellous, intracortical, and endocortical envelopes are shown in Tables 2–4. In cancellous envelope (Table 2), there were no significant differences in bone microarchitecture (BV/TV, Tb.Th and Tb.N) between patients with and without AFF. However, W.Th was significantly lower but Oc.S/BS was significantly higher in AFF patients than in non-AFF patients. There was no significant difference in any of the dynamic variables relative to tetracycline labeling between the two groups.

**Table 2:** Values of bone histomorphometric variables in cancellous envelope between BP treated patients with and without AFF.

Cancellous envelope	Non-AFF	AFF	*P*
	*n* = 32	*n* = 20	
**Structure variables**			
BV/TV (%)	13.9 (7.01)	15.2 (6.01)	.367
Tb.Th (μm)	104 (25.7)	113 (28.5)	.244
Tb.N (#/mm)	1.30 (0.364)	1.31 (0.249)	.679
T.Ar (mm^2^)	30.3 (8.95)	32.4 (12.0)	.807
B.Ar (mm^2^)	4.14 (2.12)	4.77 (2.14)	.208
BS.L (mm)	61.7 (24.2)	65.2 (21.5)	.585
**Static variables**			
W.Th (μm)	31.4 (4.11)	28.9 (4.72)	**.039**
ES/BS (%)	2.48 (1.76)	3.54 (2.77)	.142
Oc.S/BS (%)	0.345 (0.321)	0.919 (1.06)	**.005**
OV/BV (%)	0.400 (0.616)	0.805 (1.77)	.367
OS/BS (%)	2.58 (2.86)	3.94 (6.99)	.573
Ob.S/BS (%)	0.444 (0.788)	0.804 (1.47)	.885
O.Th (μm)	6.69 (3.62)	6.90 (3.32)	.520
**Dynamic variables**			
MS/BS (%)	0.849 (1.07)	1.10 (1.48)	.977
MAR (μm/d)	0.321 (0.150)	0.337 (0.177)	.878
BFR/BS (μm^3^/μm^2^/yr)	1.11 (1.70)	1.60 (2.53)	.872
BFR/BV (%/yr)	1.81 (2.84)	2.51 (4.31)	.768
Ac.f (#/yr)	0.035 (0.055)	0.052 (0.081)	.932

Data expressed as mean (SD). AFF, atypical femoral fracture; B.Ar, bone area; BS.L, bone surface length; BV/TV, bone volume per tissue volume; T.Ar, tissue area; Tb.N, trabecular number; Tb.Th, trabecular thickness.

In intracortical envelope, there was no significant difference in microarchitecture (BV/TV and Ct.Th) between patients with and without AFF (Table 3). Similar to the cancellous envelope, W.Th was significantly lower but bone resorption variables (erosion surface [ES/BS] and osteoclast surface [Oc.S/BS]) were significantly higher in AFF patients than in non-AFF patients (Table 3). In addition, none of the dynamic variables in either cortical envelope was significantly different between AFF and non-AFF patients (Tables 3 and 4).

**Table 3:** Values of bone histomorphometric variables in intracortical envelope between BP-treated patients with and without AFF.

Intracortical evelope	Non-AFF	AFF	*P*
	*n* = 32	*n* = 20	
**Static variables**			
W.Th (μm)	42.1 (6.84)	37.6 (5.91)	**.009**
ES/BS (%)	2.36 (2.44)	3.60 (2.24)	**.021**
Oc.S/BS (%)	0.433 (0.518)	0.606 (0.604)	**.048**
OV/BV (%)	0.157 (0.212)	0.183 (0.209)	.522
OS/BS (%)	5.95 (5.20)	7.38 (5.39)	.208
Ob.S/BS (%)	1.24 (1.96)	1.60 (1.96)	.335
O.Th (μm)	6.82 (2.72)	6.97 (2.94)	.910
**Dynamic variables**			
MS/BS (%)	3.22 (3.32)	2.80 (3.16)	.629
MAR (μm/d)	0.358 (0.125)	0.344 (0.125)	.769
BFR/BS (μm^3^/μm^2^/yr)	4.88 (6.48)	3.92 (5.37)	.684
BFR/BV (%/yr)	1.21 (1.66)	0.839 (0.979)	.603
Ac.f (#/yr)	0.116 (0.156)	0.113 (0.163)	.887

Data expressed as mean (SD).

**Table 4:** Values of bone histomorphometric variables in endocortical envelope between BP treated patients with and without AFF.

Endocortical envelope	Non-AFF	AFF	*P*
	*n* = 32	*n* = 20	
**Static variables**			
W.Th (μm)	36.5 (5.08)	33.6 (6.30)	.065
ES/BS (%)	3.60 (2.92)	6.57 (7.91)	.221
Oc.S/BS (%)	0.753 (0.897)	1.90 (2.55)	.213
OS/BS (%)	4.90 (4.49)	6.14 (6.59)	.693
Ob.S/BS (%)	1.13 (1.79)	1.98 (3.10)	.414
O.Th (μm)	5.92 (2.53)	4.83 (2.80)	.063
**Dynamic variables**			
MS/BS (%)	1.95 (3.07)	2.74 (3.99)	.741
MAR (μm/d)	0.290 (0.098)	0.295 (0.115)	.616
BFR/BS (μm^3^/μm^2^/yr)	2.08 (3.15)	3.16 (4.93)	.892
Ac.f (#/yr)	0.059 (0.087)	0.096 (0.148)	.834

Data expressed as mean (SD).

Table 5 shows the occurrence of no double tetracycline labels in cancellous, intracortical, endocortical envelopes and whole bone in AFF and non-AFF patients. There was no significant difference in the subjects with no double labels between AFF and non-AFF groups in any bone compartment.

**Table 5:** Absence of double tetracycline labels in AFF and non-AFF patients.

	No double labels	With double labels	*P*
Cancellous envelope			
Non-AFF	10 (31.3%)	22 (68.7%)	.561
AFF	8 (40.0%)	12 (60.0)	
Intracortical envelope			
Non-AFF	11 (34.4%)	21 (65.6)	.549
AFF	5 (25.0%)	15 (75.0%)	
Endocortical envelope			
Non-AFF	19 (59.4%)	13 (40.6%)	.781
AFF	11 (65.0%)	9 (45.0)	
Whole bone			
Non-AFF	5 (15.6%)	27 (84.4%)	.719
AFF	4 (20.0%)	16 (80.0)	

Data expressed as number (percent).

Table 6 shows the prevalence of SSBT in cancellous, intracortical, endocortical envelopes, and whole bone in both AFF and non-AFF patients. The SSBT of whole bone was determined by the Ac.f in all 3 envelopes falling within the SSBT range (as stated in Table 6). No significant difference in SSBT prevalence was found in any bone compartment between AFF and non-AFF patients.

**Table 6:** Prevalence of severely suppressed bone turnover (SSBT) in AFF and non-AFF patients.

	SSBT	Normal	*P*	Ac.f range^b^
				
Cancellous envelope				0–<0.020
Non-AFF	17 (53.1%)	15 (46.9%)	.776	
AFF	12 (60.0%)	8 (40.0%)		
Intracortical envelope				0 - <0.007
Non-AFF	8 (25.0%)	24 (75.0%)	1.000	
AFF	5 (25.0)	15 (75.0%)		
Endocortical envelope				0 - 0
Non-AFF	12 (37.5%)	20 (62.5%)	.772	
AFF	9 (45.0%)	11 (55.0)		
Whole bone^a^				
Non-AFF	4 (12.5%)	28 (87.5%)	.695	
AFF	4 (20.0%)	16 (80.0%)		

Data expressed as number (percent.

a Cancellous + intracortical + endocortical envelopes.

b Ref. ^26^.

## Discussion

In this study, we found that W.Th was significantly lower in AFF patients than in non-AFF patients in both cancellous and cortical bone, but there was no significant difference in bone microarchitecture between the 2 groups. The W.Th indicates the thickness of new bone that has refilled previously excavated bone resorption cavities.^50-53^ If the bone resorption cavities, despite their depth, are not completely refilled by new bone, it would lead to a lower W.Th associated with net bone loss, particularly with decreased Tb.Th.^50^^,^^53^ Alternatively, W.Th can also be reduced when refilling shallower resorption cavities with balanced bone resorption and formation.^51^ In this case, the lower W.Th is not accompanied by obvious bone loss. Although inhibition of bone resorption by BP does not worsen bone microarchitecture, it would reduce the depth of bone resorption, which in turn reduces the amount of newly formed bone. Reduced new bone enables the compromise of bone biomechanical properties.^54^^,^^55^ Thus, in osteoporosis patients treated for >4 yr with BPs, lower W.Th would be a risk factor for AFF.

An unexpected finding in the intracortical and cancellous envelopes of AFF patients was that, in contrast to W.Th, ES/BS and Oc.S/BS (cancellous envelope only) were increased, rather than decreased, compared with non-AFF patients. Our previous study revealed that ES/BS and Oc.S/BS in AFF patients on long-term BP therapy were similar to those in normal premenopausal women.^24^ The possible explanation is that the lengths of ES/BS and Oc.S/BS are positively associated with osteoclast number and size but do not reflect the activity and function of osteoclasts. There is evidence that BPs may reduce the depth of bone resorption but increase the number and size of osteoclasts on the bone surface.^56-60^ Weinstein et al.^59^ reported that, in early postmenopausal women, the number of osteoclasts was increased after 3 yr of alendronate treatment and approximately one-third of the osteoclasts became giant, hyper-nucleated, and detached from the bone surface, both of which may sustain the extent of erosion surface (ES/BS) and osteoclast surface (Oc.S/BS) in bone. The resorption cavities under giant osteoclasts are shallower because the bone resorption capability of these osteoclasts is reduced.^57^^,^^59^ Accordingly, BPs inhibit bone resorption by the mechanism of reducing osteoclast activity and function rather than osteoclast number.^59^^,^^61^ In this study, decreased W.Th but increased ES/BS and Oc.S/BS in AFF patients suggest that the osteoclasts in AFF patients may have lower bone resorption capability than the osteoclasts in non-AFF patients, leading to reduced W.Th.

Though a few articles using bone histomorphometry have suggested that BP-related AFF is associated with SSBT,^21^^,^^28^^,^^62^ none of these studies used well-matched BP-treated, non-AFF patients as the comparator group. The current study found no significant difference in bone formation and turnover between AFF and non-AFF patients in both cancellous and cortical bone. However, our unpublished data showed that bone formation and turnover in the BP-treated patients, both AFF and non-AFF, were significantly lower in all bone envelopes than in the age-matched (66.2 ± 5.9 yr) White osteoporotic women without receiving BP treatment.^52^ This demonstrated that the magnitude of decrease in bone formation and turnover was comparable in AFF and non-AFF patients. In this study, we defined SSBT when the Ac.f at the iliac crest fell below the lower limit of the reference interval for healthy pre-menopausal women, as we reported previously.^25^ Though absence of double tetracycline labeling is frequently found in patients with AFF, we found no significant difference in the prevalence of either no double labeling or SSBT in both cancellous and cortical bone when comparing BP-treated AFF patients to matched BP-treated non-AFF patients. Taken together, our findings could not link AFF to SSBT because a similar reduction in cancellous and cortical bone turnover occurs in both AFF and non-AFF patients after BP treatment.^29^^,^^30^^,^^63^

The current study included 32 non-AFF patients and 20 AFF patients with matched range of BP duration (4-17 yr). However, the results showed that BP duration in AFF patients was still significantly longer than that in non-AFF patients because BP duration had positive skewness in AFF patients but negative skewness in non-AFF patients. The proportion of subjects with BP duration >8 yr was significantly higher in AFF group than in non-AFF group. Accordingly, AFF patients in this study had a significantly longer duration of BP treatment than non-AFF patients, supporting the previously established idea that longer duration of BP treatment is a risk factor for AFF.^3^^,^^20^

A possible limitation of this study was that the bone biopsies were obtained from the ilium rather than region of the femur where AFFs occur. Although the pharmacological interventions may produce different effects on these 2 types of bone, the iliac bone biopsy is the best clinically accessible and the most common site used for studies on bone histomorphometry in AFF patients.^21^^,^^64^ Furthermore, obtaining a large number of biopsies from the lateral cortex of the subtrochanteric region of the femur is impractical in clinical practice, particularly from patients without AFF. Additionally, bone turnover at or near the site of AFF would be stimulated after a fracture^62^; this focal increase in bone turnover may not represent the real status of bone turnover prior to the occurrence of AFF.

In conclusion, BP-treated AFF patients had significantly lower W.Th than BP-treated non-AFF patients in both cancellous and cortical bone. The BP-treated AFF patients did not differ significantly from BP-treated non-AFF patients for bone formation, activation frequency, and MAR. Collectively, our study results suggest that AFF risk is increased in BP-treated patients with smaller young and healthy superficial bone area (indicated by lower W.Th) and more extended BP treatment.

## Author contributions

Shijing Qiu (Conceptualization, Investigation, Data curation, Formal analysis, Methodology, Writing—original draft, Writing—reviewing & editing), Ruban Dhaliwal (Conceptualization, Writing—original draft, Writing—reviewing & editing), George Devine (Data curation, Formal analysis, Writing—original draft), Elizabeth Warner (Investigation, Data curation, Methodology), and Sudhaker D. Rao (Conceptualization, Supervision, Project administration, Funding acquisition, Resources, Writing—original draft, Writing—reviewing & editing)

## Funding

The work was supported by the National Institute of Arthritis and Musculoskeletal and Skin Diseases (under Award Number AR062103).

## Conflicts of interest

None declared.

## Data availability

Data available on request. The data underlying this article will be shared on reasonable request to the corresponding author.
